# Impact of Intracranial Hypertension on Outcome of Severe Traumatic Brain Injury Pediatric Patients: A 15-Year Single Center Experience

**DOI:** 10.3390/pediatric14030042

**Published:** 2022-08-16

**Authors:** Christos Tsitsipanis, Marianna Miliaraki, Konstantinos Ntotsikas, Dimitrios Baldounis, Emmanouil Kokkinakis, George Briassoulis, Maria Venihaki, Antonios Vakis, Stavroula Ilia

**Affiliations:** 1 Department of Neurosurgery, School of Medicine, University of Crete, 70013 Heraklion, Greece; 2 Pediatric Intensive Care Unit, School of Medicine, University of Crete, 70013 Heraklion, Greece; 3 Department of Internal Medicine, Sotiria Thoracic Diseases Hospital of Athens, 11527 Athens, Greece; 4 Department of Health Sciences, National and Kapodistrian University of Athens, 11527 Athens, Greece; 5 Department of Clinical Chemistry, School of Medicine, University of Crete, 70013 Heraklion, Greece

**Keywords:** ICP monitoring, intracranial pressure, brain injury, pediatric, trauma

## Abstract

Background: Intracranial hypertension (IC-HTN) is significantly associated with higher risk for an unfavorable outcome in pediatric trauma. Intracranial pressure (ICP) monitoring is widely becoming a standard of neurocritical care for children. Methods: The present study was designed to evaluate influences of IC-HTN on clinical outcomes of pediatric TBI patients. Demographic, injury severity, radiologic characteristics were used as possible predictors of IC-HTN or of functional outcome. Results: A total of 118 pediatric intensive care unit (PICU) patients with severe TBI (sTBI) were included. Among sTBI cases, patients with GCS < 5 had significantly higher risk for IC-HTN and for mortality. Moreover, there was a statistically significant positive correlation between IC-HTN and severity scoring systems. Kaplan–Meier analysis determined a significant difference for good recovery among patients who had no ICP elevations, compared to those who had at least one episode of IC-HTN (log-rank chi-square = 11.16, *p* = 0.001). A multivariable predictive logistic regression analysis distinguished the ICP-monitored patients at risk for developing IC-HTN. The model finally revealed that higher ISS and Helsinki CT score increased the odds for developing IC-HTN (*p* < 0.05). Conclusion: The present study highlights the importance of ICP-guided clinical practices, which may lead to increasing percentages of good recovery for children.

## 1. Introduction

Severe traumatic brain injury (TBI) always remains a significant cause of severe disability and mortality in children, despite advances in pediatric neurocritical care. Elevated intracranial pressure (ICP) seems to significantly contribute to unfavorable outcomes, while recent trends highlight the importance of aggressive therapy, in order to avoid secondary neurological injury [1]. Severe TBI (sTBI) is usually followed by intracranial hypertension (IC-HTN) and reduction of cerebral perfusion pressure, which could lead to significant morbidity, especially for pediatric patients with limited cerebral autoregulation reserves [2]. Therefore, ICP monitoring could allow intensivists to optimize cerebral perfusion and prevent secondary brain injury, through objective and graded use of suitable therapies [3]. While brain oxygenation and metabolism monitoring may provide more future opportunities for critical interventions in severe TBI patients, ICP monitoring is currently the mainstay in early detection of the deteriorating critically injured patient [4], with recent reports pointing toward lower risk of in-hospital mortality for ICP-monitored patients [5,6]. However, the benefits and effectiveness of ICP monitoring still remain controversial with wide variability in adherence to ICP-guided practice, especially for the pediatric population [7,8,9]. Mortality rates currently reported for pediatric intensive care unit (PICU) TBI patients can be as high as 20% [10], which emphasizes the imperative need of establishing specific guidelines, in order to improve the quality of health care delivery and to optimize patient outcomes [11].

Intracranial hypertension (IC-HTN) in the pediatric population is generally defined by an ICP exceeding 20 mmHg, although slightly lower thresholds (>15 mmHg) are generally accepted for younger children [12]. When treating pediatric brain trauma patients, certain anatomic and physiologic differences between children and adults should be taken into account. The pediatric scalp is relatively heavier and highly vascularized, leading to higher risk of lethal blood loss. Moreover, due to higher plasticity and deformity, fewer mass lesions but more white matter shear lesions may occur, while anatomic differences in myelination are pronounced during progressing development, accounting for different absorption of traumatic forces in younger children [13,14]. The most significant secondary complication following sTBI is the emergence of cerebral edema, and because of the developing biological processes of autoregulation for restoring brain blood supply, the vulnerability of children to cerebral hyperemia and intracranial hypertension seems to be critical [15]. Both cytotoxic and vasogenic edema may occur, which can be further exacerbated by physiologic derangements, such as hypoxia, hypotension, or hyperthermia [16]. Therefore, ICP normalization seems to be of major importance in order to optimize oxygen delivery and cerebral perfusion, and prevent cerebral herniation [17]. This could possibly restrain secondary disastrous cascades including neuronal damage, neurotransmitter dysfunction, impaired cellular metabolism, cerebral hypoxia or inflammation in the central nervous system [18].

Current treatment strategies for IC-HTN in children, similarly to adults, follow the principles of the Monro–Kellie doctrine on balancing volumes of cerebral blood and cerebrospinal fluid (CSF) [19], and rely on recently updated pediatric therapeutic algorithms. These include first-tier conservative measures, including sedatives, paralytics, and hyperosmolar therapies [20], followed by controversial second or last-tier therapies, such as barbiturates or rescue surgical techniques for decompressive craniectomy in cases of refractory IC-HTN [21,22,23].

Several studies support coordinated implementation of practice guidelines, including adherence to ICP monitoring, in order to improve outcomes of TBI patients [24,25]. Invasive fiber optic intraparenchymal ICP monitors or even external ventricular drains (EVD) continue to be considered essential aids for early goal-directed therapy [26], while the significance of multimodal invasive on noninvasive neuromonitoring practices in children is currently under investigation [27,28]. With regard to complications, intraparenchymal devices are rarely linked to serious infectious or hemorrhagic side effects, in contrast to EVDs, which have principally been associated with ventriculitis or intracerebral hemorrhage to a much greater extent [29].

Prognostic information would be a useful additional aid for caregivers, in order to guide clinical decisions concerning children with life-threatening injuries. In pediatric patients, the Glasgow Coma Scale (GCS) is primarily used as an assessment tool for predicting TBI severity, while a GCS ≤ 8 after injury along with abnormal brain radiographic imaging are usually considered as the main indications for ICP monitoring [30]. Based on CT imaging characteristics, several classification systems have been designed, including the Marshall and Rotterdam scores, which were developed to predict the severity of TBI, or the Helsinki CT scoring system, which seems to have the ability to predict long-term outcome [31].

Given the limited number of studies regarding children, the main objective of the present study is to evaluate influences of ICP monitoring and of IC-HTN on clinical outcomes of pediatric TBI patients, based on certain clinical and radiological characteristics.

## 2. Materials and Methods

### 2.1. Patients

A retrospective analysis of medical records regarding 154 PICU neurocritical TBI patients was conducted during the study period (January 2005–December 2021). Admission demographics, clinical parameters, therapeutic interventions, and radiological (computed tomography and magnetic resonance imaging) findings were recorded. Eligible for enrollment in the study were sTBI pediatric cases (GCS 3–8) of different age groups (infants: 0–2 years old, preschoolers: 3–5 years old, schoolers: 6–9 years old, adolescents: 10–18 years old). Severity of injury was also assessed through Injury Severity Score (ISS), Pediatric Logistic Organ Dysfunction (PELOD), and Pediatric Risk of Mortality (PRISM) scores, along with computed tomography (CT) scoring systems (Marshall, Rotterdam and Helsinki scale scores) assessing the worst CT scan within the first 24 h after admission. On the basis of imaging findings, diagnoses were subtyped into five main categories: (1) extradural hematoma, (2) subdural/intraparenchymal/intraventricular hematoma, (3) diffuse brain edema, 4) diffuse axonal injury, (5) hydrocephalus or other findings. Possible prognostic radiologic findings were also recorded (epidural or subdural hematoma, subarachnoid hemorrhage, intracerebral hematoma, midline shift, compressed or obliterated cisterns with cerebral edema, diffuse axonal injury, depressed skull fractures).

The decision for ICP monitoring was based on neurosurgical and PICU decisions after evaluating TBI severity, the need for neurosurgical interventions, or radiographic brain imaging, and taking into account the high inter-hospital variations in the use of ICP monitoring. Monitoring was performed through the insertion of available intraparenchymal ICP catheters (Camino^®^, IntegraTM LifeSciences, Plainsboro, NJ, USA; Raumedic^®^ AG, Helmbrechts, Germany). Patients who developed IC-HTN comprised the study group, while patients who had normal ICP values were assigned into the control group. Treatment of IC-HTN in sTBI patients was generally guided by the Pediatric Critical Care Medicine (PCCM) guidelines, recommending treatment of a sustained ICP > 20 mmHg for at least 5 min, through many years of evidence [17,21,32,33]. Mechanically ventilated patients in the sTBI group received sedative and analgesic medications, along with muscle relaxants in case of IC-HTN despite adequate sedation. Finally, therapeutic interventions in cases of persistent intracranial hypertension, such as osmotherapy, or second-tier therapies were recorded for statistical correlations. Duration of mechanical ventilation (MV), PICU length of stay (LOS) and Glasgow Outcome Scale (GOS) at 6 months were also recorded.

### 2.2. Primary and Secondary Endpoints of the Study

The primary outcome measures of the study were IC-HTN correlations with certain independent clinical variables, such as age, GCS, severity scores (ISS, PELOD and PRISM scores), or radiological findings (Marshall, Rotterdam and Helsinki CT scores), along with mortality or poor neurological outcomes. Secondary endpoints of the study were possible outcome benefits of implementing ICP-based protocols, approximated through PICU length of stay (LOS), or functional neurologic outcome (GOS).

### 2.3. Statistical Analyses

Statistical analyses were conducted through SPSS software for Windows (IBM SPSS Statistics, Chicago, IL, USA, version 25). Summary statistics for quantitative variables are presented as mean with standard deviation (normal distribution), and median values with interquartile ranges (non-normal distribution). Categorical variables are summarized as proportions. Bivariable analyses required unpaired, 2-tailed Student’s *t*-test for quantitative variables in parametric comparisons, or Mann–Whitney test in non-parametric comparisons, and χ^2^ test or Fisher’s exact test for categorical variables, as appropriate. Statistical significance was set at a *p*-value of less than 0.05 for all analyses. Variables with statistically significant differences between them were used as covariates into adjusted multivariable logistic regression models using a stepwise selection process, in order to assess possible correlations of variables independently associated with IC-HTN, mortality or disability. Receiver operator characteristic (ROC) curve analyses were performed in order to tract significant variables affecting the outcome. The probability of recovery after IC-HTN at different time points was estimated by using Kaplan–Meier curve. Log-rank test was used to compare the average recovery time of patients with respect to clinical decisions.

## 3. Results

**Figure 1:**
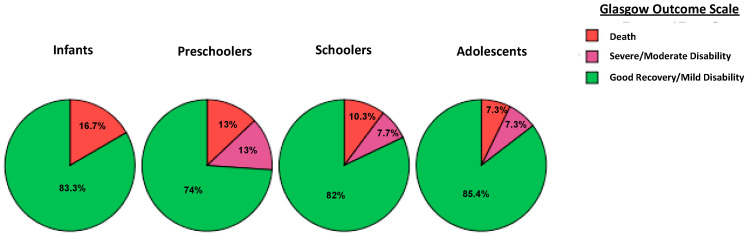
Frequencies of outcomes by Glasgow Outcome Scale (GOS) among different age groups.

One hundred and eighteen (76%) out of 154 pediatric patients enrolled in the study had sTBI. Boys accounted for 73% and girls for 27%, and the mean age of the patients was 8.5 ± 5 years. Traumatic outcomes differed among different age groups (Figure 1). Initial neurosurgical procedures were performed in 42% (n = 64) of children, depending on radiological findings. Among severe TBI cases, 66% (78 out of 118) underwent ICP monitoring, while a subgroup of children (40%) with a reassuring initial brain CT scan was not monitored. Of the 24 patients (20%) who did not undergo ICP monitoring despite abnormal CT findings, the primary causes were decisions for clinical surveillance or moribund status. Among non-ICP monitored patients, one death occurred. However, the implementation of ICP-based protocols in our PICU was increased by 15% within the last 5 years. Univariate analyses of ICP monitoring, IC-HTN and mortality with regard to the clinical parameters of the study are shown in Table 1 and Table 2. Patients in the ICP-monitored group had no higher probability of undergoing neurosurgical procedures compared to non-ICP monitored patients (OR = 1.4; 95% CI: 0.62–4.1; *p* = 0.245).

**Figure 2:**
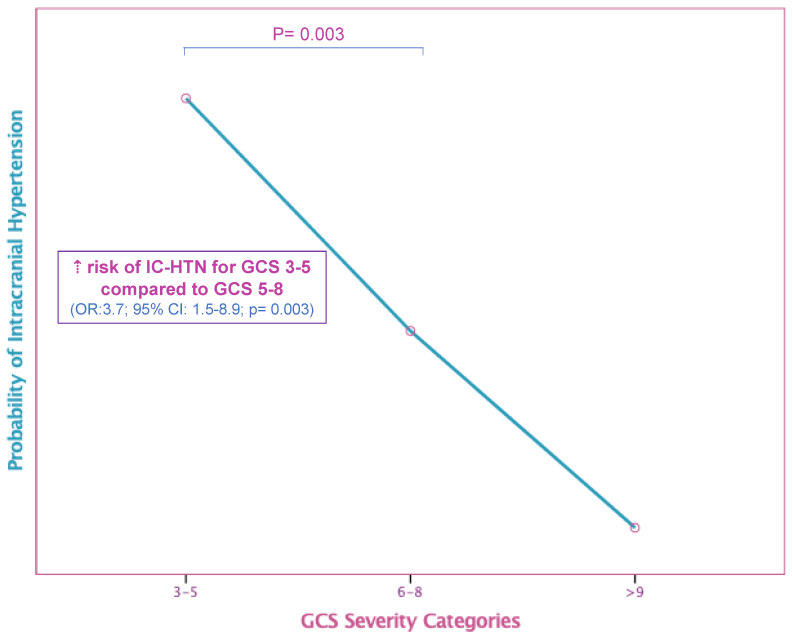
Higher risk of intracranial hypertension (IC-HTN) for severe traumatic brain injury (TBI) cases based on Glasgow Coma Scale (GCS).

**Figure 3:**
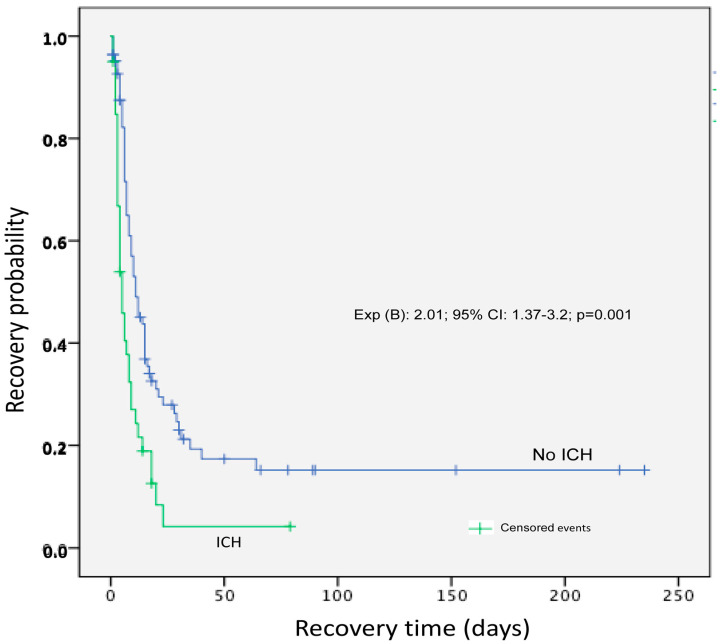
Kaplan–Meier (KM) curve showing recovery probability for severely injured patients with intracranial hypertension. Censored events shown by vertical hash marks, corresponding to alive hospital discharges, and in-hospital deaths recorded as events. Although not a significant predictor of in-hospital mortality in multivariate regression analysis, KM product-limit estimator showed a significant difference for an unfavorable outcome for those who had intracranial hypertension (log rank chi-square = 11.16, *p* = 0.001).

Regarding the IC-HTN cases and their possible prognostic determinants, the present study found that 55% (43/78) of ICP-monitored patients received ICP-directed therapy for at least one episode of IC-HTN. As expected, children in the ICP monitored group had higher severity scores (ISS, PRISM, PELOD) and lower GCS upon admission. Severe TBI cases presenting with a GCS of 3–5 were linked to a 30% higher risk of developing IC-HTN (*p* = 0.003, Figure 2). Moreover, there was a statistically significant positive correlation between IC-HTN and severity scoring systems, but no significant IC-HTN correlation was found with regard to different age groups. In the elevated ICP group of patients, unfavorable outcomes based on GOS escalated to 37% (*p* < 0.001) (Table 2). As expected, PICU LOS and MV duration were significantly higher in the ICP-monitored group (*p* < 0.001). However, although not reaching statistical significance, a positive trend of favorable outcomes in sTBI cases was noted within the last 5 years (88% of children with good recovery vs. 78% in previous years, OR = 2.07; 95% CI: 0.7–5.8; *p* = 0.12), after more active implementation of currently accepted ICP monitor-based pediatric protocols. A multivariable predictive logistic regression analysis distinguished the ICP-monitored patients at risk for developing IC-HTN. Through a stepwise selection process the model finally revealed that higher ISS and Helsinki CT score increased the odds for developing IC-HTN (*p* < 0.05, Table 3). Kaplan–Meier analysis determined a significant difference for good recovery among patients who had no ICP elevations, compared to those who had at least one episode of IC-HTN (log-rank chi-square = 11.16, *p* = 0.001) (Figure 3).

**Figure 4:**
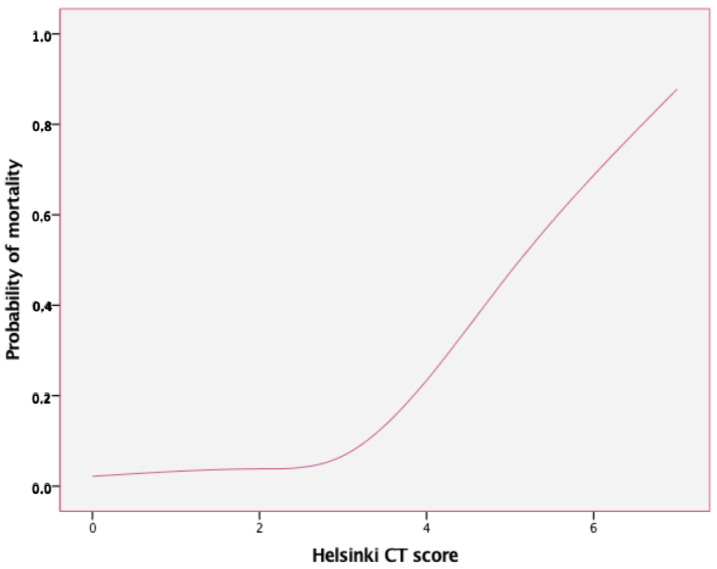
Prediction of mortality through Helsinki CT score.

As far as radiologic imaging is concerned, 58% of sTBI patients had abnormal brain imaging based on Marshall, Rotterdam, or Helsinki criteria. On the other hand, 15.6% of patients with an initial reassuring brain CT eventually developed intracranial hypertension, while 93% of these cases were adequately controlled with first-tier therapies. Regarding the connection of IC-HTN with abnormal CT findings, Pearson’s chi-square revealed a statistically significant risk of IC-HTN for those patients having higher Marshall, Rotterdam, or Helsinki CT scores (OR = 3.9, 6.2, and 6.4 respectively, *p* < 0.001), but on linear regression models the Rotterdam and Helsinki CT scores exhibited better predictive ability. With regard to the association between imaging findings and mortality, only the Helsinki CT score proved to be a statistically significant independent factor for an unfavorable outcome (B = 0.33; 95% CI: 0.06–0.2, *p* = 0.001). Mortality probability based on Helsinki CT score is shown in Figure 4. Among patients undergoing mass-effect neurosurgical procedures (type V according to Marshall CT scale), no association with subsequent intracranial hypertension or mortality was found, whereas positive outcomes in terms of disability were recorded in 81% of cases. Additionally, the prognostic value of certain radiologic findings was highlighted by the fact that midline shift and compressed or obliterated cisterns showed a statistically significant correlation with mortality, whereas subarachnoid hemorrhage and intracerebral hemorrhage were significantly linked to severe disability (*p* < 0.03).

**Figure 5:**
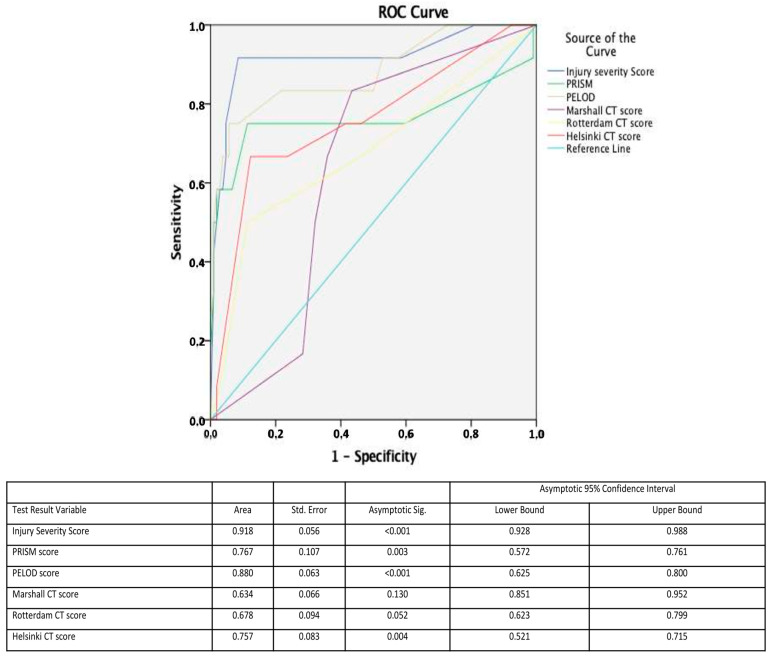
Receiver operator curve (ROC) analysis showing higher area under the curve for ISS and PELOD score. Among the CT scoring systems, the Helsinki CT score displayed the highest performance.

Mortality rates in sTBI patients reached 10.2%, while three preschoolers (2.5%) who sustained abusive head trauma eventually died. With reference to possible clinical prognostic factors for mortality, multiple analyses for the different GCS score subgroups, and after adjusting for age and sex, revealed that ICP monitoring was not found to be a significant determinant of survival, probably reflecting the severity of the primary insult. Overall mortality was higher in the IC-HTN group (23% vs. 2.5%, *p* = 0.001). Among sTBI cases, patients with GCS < 5 had a significantly higher risk for IC-HTN (OR = 3.8; 95% CI 1.5–9.2; *p* = 0.003) and for mortality (OR = 11.5; 95% CI 3.4–18.6; *p* < 0.001). As expected, age did not prove to be a significant predictor of mortality, but the probability of an unfavorable outcome was positively correlated with higher values of prognostic scoring systems (ISS, PRISM and PELOD). With regard to severe disability and after controlling for all other factors, ISS and PELOD score were also independent predictors of unfavorable outcomes (*p* < 0.009, Table 3). Multivariable logistic regression analyses using stepwise forward selection processes yielded a predictive model for mortality, which finally included ISS and PELOD scores (*p* < 0.005). In predicting mortality, the area under the curve (ROC) for ISS and PELOD score was 0.918 (95% CI: 0.93–0.99; *p* < 0.001) and 0.88 (95% CI: 0.63–0.8; *p* < 0.001) respectively. Among the CT scoring systems, the Helsinki CT score displayed the highest performance (AUC 0.76, 95% CI: 0.52–0.715; *p* = 0.004) (Figure 5).

Regarding first-tier interventions in patients with IC-HTN, osmotic agents such as mannitol or hypertonic saline, in addition to maximum sedation/paralytics were found to control ICP in a high percentage of patients (50%). Second-tier interventions for refractory ICP including barbiturates and secondary decompressive craniectomy were used in 37.5% and 25% of cases, respectively. Among those patients who underwent secondary decompressive craniectomy, 67% had favorable functional outcomes based on 6-month GOS. Initial neurosurgical procedures were associated with higher risk for second-tier interventions (*p* = 0.001). On multivariable logistic regression analyses, anisocoria and Rotterdam CT score yielded the best predictive model regarding the risk for a secondary decompressive craniectomy (*p* = 0.001).

A low percentage (3.4%) of ICP monitoring related hemorrhagic complications was found in the present study, principally concerning small parenchymal hemorrhages around the area of the catheter placement not requiring neurosurgical interventions, but no ICP-catheter-related infectious complications were observed.

## 4. Discussion

Even though extensive literature has recently been published, the potential benefit of invasive ICP monitoring still remains controversial, despite high percentages of IC-HTN in pediatric sTBI cases, or the generally accepted association between IC-HTN and worse neurologic outcomes [3,34]. The present study evaluated the influences of IC-HTN on the outcome of sTBI pediatric patients, and reports a high percentage of nearly 66% of children who were managed through ICP-guided protocols. Even though lower rates of IC-HTN were found compared to previous reports [3,29], an initial GCS score < 5 was associated with higher risk for intracranial hypertension and for an unfavorable outcome based on 6-month GOS. The main findings of the study highlight the importance of ICP-guided clinical practices, which may not reduce the risk of mortality, but lead to increasing percentages of good recovery for children. Recent meta-analyses have already reported similar results in adults, proposing consensus-based management algorithms for IC-HTN [35,36]. Contrary to an initial hypothesis, no specific GCS group of ICP-monitored patients exhibited any mortality benefit, although another study has reported that ICP monitoring seems to be associated with a reduction in mortality only for patients with a GCS score of 3 [30]. Controversial benefits regarding survival in ICP-monitored patients have been attributed to aggressive treatment interventions and longer periods of sedation (1). Researchers have repeatedly reported the difficulties of performing prospective randomized clinical trials to evaluate the effect of ICP monitoring on outcomes of PICU patients, since there are ethical concerns of not monitoring ICP in the control group of patients. Major pediatric TBI guidelines are currently based on ICP measurements, while ICP monitoring has been established as a standard of care for sTBI [30,37,38]. Moreover, studies show that the implementation of evidence-based TBI treatment algorithms are associated with improved outcomes, highlighting the importance of future precise ICP-based guidelines in order to reduce practice disparity among TBI patients [39]. A low percentage (3.4%) of ICP monitoring-related hemorrhagic complications was found in the present study, similar to the published literature [34,40].

With regard to second-tier interventions, a high percentage of 67% of those patients who underwent decompressive craniectomy had a favorable functional outcome. This is in accordance to recent studies both in children and adults showing that patients with refractory IC-HTN can achieve a good neurologic outcome after secondary decompressive craniectomy [36,41,42,43], while current reports highlight the importance of early and aggressive treatment algorithms, especially for pediatric TBI patients, who have higher recovery rates compared to adults [13]. However, other detailed reviews in adults highlight the significantly reduced mortality, but also the increased proportions of severely disabled patients in the decompressive craniectomy group of patients, when compared to conservatively treated medical groups.

Several studies also evaluate whether overreliance on ICP-monitoring may prolong intensive care stay, since the traumatic injury itself possibly triggers biologic reactions, such as excitotoxic injury or autoregulatory failure, exacerbating the initial insult [4,44]. As expected, mean PICU LOS and mechanical ventilation periods are longer for pediatric patients undergoing ICP monitoring. This long-term hospitalization may reflect the severity of the primary insult, the prolonged duration of clinical practices in order to treat ICP elevations, or the higher probability for neurosurgical interventions in sTBI, which in turn could account for significant complications as well [5].

Despite advances in diagnostic and therapeutic clinical practices for sTBI, the clinical determinants of outcome remain unclear, especially for pediatric patients. The categorization of patients based on the injury severity level is still challenging, with the need for more precise prognostic tools [6]. Recent meta-analyses have already shown that patients with a better GCS and normal ICP, or those responding to ICP lowering treatments, are more likely to have a higher potential for recovery [45]. Our results are in agreement with other studies reporting an insignificant predictive association of IC-HTN with GCS, age, ISS or pupil reactivity [3]. Despite the clinical descriptive value of GCS, its adequacy as a grading tool of TBI severity has been questioned, since it does not provide any structural information regarding intracranial lesions. Moreover, recent reports in pediatric trauma patients have revealed that an elevated ISS is valuable in prediction of impaired functional independence, while both PRISM and PELOD scores were reliable prediction tools for mortality [46,47,48]. Additionally, even though it has been suggested that certain anatomical or physiological differences in younger children’s metabolism might make them more prone to severe primary or secondary brain injury compared to adults, the prognostic value of age as a predictor of poor outcome has not yet been confirmed, similarly to our findings [49,50]. An interesting recent report has connected the clinical or prognostic differentiations of TBI patients with anatomic variations among ethnicities or sexes or with age-related atrophy, which could account for time-delays in symptomatology of IC-HTN in certain individuals [51].

On the other side of the coin, there are few prognostic models based on radiologic findings validated for the pediatric TBI population [52]. Brain scanning through non-contrast or cerebral perfusion computed tomography (CT) may not only be used for diagnostic screening of intracranial injuries requiring neurosurgical interventions, but are also beginning to be essential tools for prognostic stratification of patients based on certain radiological features or standardized imaging scores [53,54,55]. In the present study, the Helsinki CT classification, along with the Rotterdam and Marshall CT scores were assessed using initial CT scans. Moreover, certain CT findings were found closely predictive of IC-HTN. Similarly to our results, recent studies report the better performance of the more detailed Helsinki CT score in predicting unfavorable outcomes [52,56,57,58,59]. Based on our records, anisocoria and higher Rotterdam CT scores yielded the best predictive model regarding the risk for last-tier interventions, while a validated pediatric mortality model based on Rotterdam CT score has recently been reported as accurate in children with moderate to severe TBI, and higher Rotterdam scores have been associated with worse survival in children compared to adults [60]. Further prospective studies with different clinical and radiological multivariable combinations could lead to novel IC-HTN predictive models and determination of patients who would benefit from ICP-guided therapies.

## 5. Conclusions

In conclusion, ICP monitoring may guide critical therapeutic decisions in order to increase favorable functional outcomes in pediatric brain-injured patients. Our findings support the safety of use of ICP monitors in pediatric patients who meet the criteria for severe brain injury. The present study revealed that IC-HTN was significantly connected with higher risk for an unfavorable outcome. In fact, this relationship seems to be associated with certain clinical findings, such as lower GCS at presentation, or higher severity scoring systems (ISS, PELOD), along with abnormal radiological algorithms (Helsinki CT score) in pediatric neurotrauma patients. Larger randomized controlled trials are necessary to further identify specific ICP monitoring indications and to standardize ICP thresholds. This will provide clarity on the appropriate pediatric neurocritical care protocols that need to be instituted.

## 6. Limitations

An important limitation of the present study is the retrospective design for data acquisition and the lack of prospective information regarding TBI patients. In addition, it was carried out in a single PICU neurotrauma center, with a lot of patients from nearby areas with multiple levels of severity of injury. Over the 15-year time span of the study, changes in TBI guidelines have also occurred leading to clinical practice alterations. However, given the paucity of literature on ICP monitoring in children, the present analysis might provide a scientific step for future studies and might motivate pediatric intensivists to focus their clinical decisions on specific goals guided by ICP monitoring in order to avoid IC-HTN.

## Figures and Tables

**Table 1:** Descriptive statistics and univariate comparisons.

Variable	ICP Monitoring (n = 78)	No ICP Monitoring (n = 40)	*p*-Value
**Sex, n (%)**			NS
Males	55 (70.5)	31 (77.5)	
Females	23 (29.5)	9 (22.5)	
**Age (mean, SD)**	10.1 (5.5)	8.2 (5)	**0.001**
**Severity of illness measures**			
GCS score, median (IQR)	8 (3–8)	9.5 (8–15)	**0.001**
ISS score, median (IQR)	14 (10–20)	7.5 (5–14)	**0.003**
PRISM score, median (IQR)	11 (2–21)	2 (0–11)	**0.013**
PELOD score, median (IQR)	11 (6–12)	2 (0–11)	**0.001**
**Imaging findings**			
Marshall CT, n (%)			NS
I + II	43 (55)	28 (70)	
III + IV	13 (17)	1 (2.5)	
V + VI	22 (28)	11 (27.5)	
Rotterdam CT, n (%)			**0.001**
1	35 (45)	25 (62.5)	
2	26 (33)	13 (32.5)	
3	13 (17)	2 (5)	
4	4 (5)	0 (0)	
Helsinki CT score (median, IQR)	2 (0–5)	0 (0–2)	**0.001**
**Hospital Outcomes**			
PICU LOS, median (IQR)	10 (5–18)	5 (2–11)	**0.001**
MV days, median (IQR)	7 (4–12)	2 (0–4)	**0.001**
**Glasgow Outcome Score, n (%)**			**0.002**
Good Outcome/Moderate disability	60 (77)	37 (92.5)	
Severe disability/Vegetative State	7 (9)	2 (5)	
Death	11 (14)	1 (2.5)	

Statistical significance was defined according to the 95% confidence level. IQR = interquartile range, NS = non-significant.

**Table 2:** Descriptive statistics by outcome group.

Variable	ICH (n = 43)	No ICH (n = 75)	*p*-Value	Survivors (n = 106)	Non-Survivors (n = 12)	*p*-Value
**Sex, n (%)**			NS			NS
Males	29 (67.5)	57 (76)		77 (72.6)	9 (75)	
Females	14 (32.5)	18 (24)		29 (27.4)	3 (25)	
**Age (mean, SD)**	10 (5.8)	9 (5.2)	NS	9 (5.5)	7.5 (4)	NS
**Severity of illness measures**						
GCS score, median (IQR)	7 (3–8)	9.5 (8–13)	0.001	8 (8–12)	3 (3–8)	**0.001**
ISS score, median (IQR)	16 (14–30)	10 (5–14)	0.001	10 (5–14)	40 (20–75)	**0.001**
PRISM score, median (IQR)	12.5 (2.0–22)	2 (1–11)	0.001	2 (2–11)	25 (12–35)	**0.001**
PELOD score, median (IQR)	11.5 (8–16)	6 (2–11)	0.001	6 (2–11)	21 (12–32)	**0.001**
**Imaging findings**						
Marshall CT, n (%)			NS			NS
I + II	21 (49)	50 (66.7)		67 (63)	4 (33)	
III + IV	8 (18.5)	6 (8)		8 (8)	6 (50)	
V + VI	14 (32.5)	19 (25.3)		31 (29)	2 (17)	
Rotterdam CT, n (%)			0.001			**0.030**
1	13 (30.3)	47 (62.7)		56 (52.8)	4 (33)	
2	17 (39.5)	22 (29.3)		37 (35)	2 (17)	
3	11 (25.6)	4 (5.3)		10 (9.4)	5 (42)	
4	2 (4.6)	2 (2.7)		3 (2.8)	1 (8)	
Helsinki CT score (median, IQR)	2 (0–5)	0 (0–2)	0.001	0 (0–2)	5 (0–5)	**0.001**
**Hospital Outcomes**						
PICU LOS, median (IQR)	10 (4–20)	5 (4–13)	0.015	8 (4–15)	4 (2–15)	NS
MV days, median (IQR)	7 (4–16)	3 (3–8)	0.005	4 (1–8)	5 (2–14)	NS
**GOS, n (%)**			0.005			**0.001**
Good Outcome/ Moderate disability	27 (63)	70 (93.3)		97 (91.5)	0 (0)	
Severe disability/ Vegetative State	6 (14)	3 (4)		9 (8.5)	0 (0)	
Death	10 (23)	2 (2.7)		0 (0)	12 (100)	

Statistical significance was defined according to the 95% confidence level. IQR = interquartile range, NS = non-significant.

**Table 3:** Multivariate linear regression of potential predictors related to IC-HTN and unfavorable outcome for sTBI pediatric patients.

	Intracranial Hypertension			Unfavorable Outcome		
	*p*-Value	Odds Ratio	95% CI	*p*-Value	Odds Ratio	95% CI
**Age (1 year)**	0.678	0.981	0.89–1.07	0.587	1.051	0.88–1.25
**Female Sex**	0.447	0.677	0.25–1.85	0.189	4.166	0.5–35
**GCS**	0.359	1.155	0.85–1.57	0.677	0.878	0.48–1.6
**ISS**	0.039	0.958	0.89–0.98	**0.001**	0.842	0.76–0.94
**PRISM score (>20)**	0.650	0.984	0.91–1.05	0.120	1.122	0.97–1.3
**PELOD score (>20)**	0.806	0.994	0.95–1.04	**0.009**	0.736	0.58–0.93
**Marshall CT score**	0.491	0.896	0.65–1.22	0.301	0.735	0.41–1.3
**Rotterdam CT score**	0.563	1.38	0.46–4.15	0.121	5.761	0.63–52.8
**Helsinki CT score**	0.048	0.711	0.5–0.91	0.141	0.645	0.36–1.16
**Intracranial Hypertension**	-	-	-	0.297	2.597	0.43–15.6

Multivariate linear regression describing possible associations with mortality. Statistical significance was defined according to the 95% confidence level.

## Data Availability

The data presented in the study are all contained within this article.
